# Prognostic value of receptor tyrosine kinases in malignant melanoma patients: A systematic review and meta-analysis of immunohistochemistry

**DOI:** 10.3389/fonc.2022.819051

**Published:** 2022-09-23

**Authors:** Xuan Lei, Yiming Zhang, Lianghao Mao, Pan Jiang, Yumeng Huang, Jia Gu, Ningzheng Tai

**Affiliations:** ^1^ Department of Burns and Plastic Surgery, Affiliated Hospital of Jiangsu University, Zhenjiang, China; ^2^ Department of Orthopedics, Affiliated Hospital of Jiangsu University, Zhenjiang, China

**Keywords:** receptor tyrosine kinases, malignant melanoma, prognostic value, survival analysis, clinicopathological features

## Abstract

**Background:**

Substantial evidence suggests that receptor tyrosine kinases (RTKs) are overexpressed in tumors; however, few studies have focused on the prognostic value of RTKs in melanoma.

**Objectives:**

The objective of this study is to evaluate the association between overexpression of RTKs and survival in melanoma patients based on immunohistochemistry (IHC) analysis.

**Methods:**

Our review is registered on PROSPERO (http://www.crd.york.ac.uk/PROSPERO), registration number CRD42021261460. Seven databases were searched, and data were extracted. We used IHC to measure the association between overexpression of RTKs and overall survival (OS), disease-free survival (DFS), progression-free survival (PFS), and clinicopathology in melanoma patients. Pooled analysis was conducted to assess the differences between Hazard Ratios along with 95% confidence intervals.

**Results:**

Of 5,508 publications examined following the database search, 23 publications were included in this study, which included data from a total of 2,072 patients. Vascular endothelial growth factor receptor 2 (VEGF-R2) overexpression was associated with worse OS and DFS in melanoma. Furthermore, there was an association between OS and the expression of several RTKs, including epidermal growth factor receptor (EGFR), mesenchymal-epithelial transition factor (MET), vascular endothelial growth factor receptor 1 (VEGF-R1), and insulin-like growth factor 1 receptor (IGF-1R). There were no significant correlations between EGFR overexpression and worse DFS or PFS. EGFR overexpression was associated with worse OS cutaneous and nasal melanoma, but not uveal melanoma. However, MET overexpression was related to worse OS in both cutaneous and uveal melanoma. Furthermore, EGFR overexpression was associated with a worse OS in Europe compared to other geographic areas. Moreover, EGFR and MET overexpression showed significant prognostic value in patients with the cut-off “≥10% staining”.

**Conclusions:**

Our findings build concrete evidence that overexpression of RTKs is associated with poor prognosis and clinicopathology in melanoma, highlighting RTK expression has the potential to inform individualized combination therapies and accurate prognostic evaluation.

## Introduction

Malignant melanoma is a type of skin tumor with a high mortality rate. If not detected early, melanoma will deteriorate and metastasize. Malignant melanoma most frequently occurs in males aged 50–70 years, although the incidence of malignant melanoma in young people, especially females, has increased in recent years (1). The advent of immunotherapy and targeted therapy for melanoma, such as anti-programmed death ligand 1 (PD-L1) and cytotoxic T-lymphocyte associated protein 4 (CTLA-4), has improved the survival rate of melanoma patients. Despite these therapeutic advances, patients with advanced malignant melanoma often develop drug resistance. Once distant metastasis occurs, the sustained response rate to drug therapy is only about 30% (2). Therefore, it is essential to further study melanoma pathogenesis as well as identify new biomarkers and combination treatment options to effectively treat this disease.

Receptor tyrosine kinases (RTKs) are single transmembrane receptors that participate in the development and progression of a variety of tumors. In solid tumors, overexpression or mutations of RTKs promotes the malignant biological behavior of tumor cells. Additionally, RTK overexpression is closely related to the maintenance of tumor stemness, drug resistance, recurrence, and high-metastasis rate (3–6). Some RTKs, such as epidermal growth factor receptor (EGFR) and vascular endothelial growth factor receptor (VEGFR), may represent potential biomarkers that can assist in the prognostic evaluation and inform treatment options. Faião-Flores et al. demonstrated receptor tyrosine kinase-like orphan receptor 1/2 (ROR1/2) and insulin-like growth factor 1 receptor (IGF-1R) signaling were critical pathways that participated in the escape of advanced uveal melanoma from MEK inhibition (7). Some small molecule tyrosine kinase inhibitors (TKIs) targeting carcinogenic-related RTKs have been put into clinical trials (8–10). However, it is still necessary to explore the value of RTKs as a prognostic tool, which can lead to accurate diagnosis and inform individualized treatment regimens. In some cancers, a number of RTKs, including EGFR or VEGFR, have been demonstrated as prognostic markers and there are targeting drugs for individualized therapy. However, it is still unclear which RTKs may represent prognostic biomarkers in melanoma as there is minimal evidence from comprehensive analysis to prove it. The exploration of carcinogenic RTKs has become a trendy field in cancer research. Deciphering the prognostic value of RTKs from a comprehensive analysis can provide substantial evidence for clinical survival estimation and inform the use of individualized, combined therapies especially for patients with advanced melanoma.

Because substantial evidence suggests that RTKs are overexpressed in tumors; however, few studies have focused on the prognostic value of RTKs in melanoma. To determine the prognostic value of RTKs, we systematically evaluate the association between overexpression of RTKs and clinicopathological features in patients with malignant melanoma.

## Materials and methods

This systematic review and meta-analysis followed the Preferred Reporting Items for Systematic Reviews and Meta-Analyses (PRISMA) guidelines and checklist. This study was preregistered on PROSPERO (https://www.crd.york.ac.uk/PROSPERO/) under number CRD42021261460.

### Search strategy

Three independent reviewers (XL, YZ, LM) searched seven databases: PubMed, Cochrane, EBSCOhost, Embase, Ovid, ScienceDirect, and Web of Science without language restriction on 1^st^ August 2021. Our search keywords were: “Melanoma” AND [“Receptor Tyrosine Kinases” OR “EGFR (Epidermal Growth Factor Receptor)” OR “IGFR (Insulin-Like Growth Factor Receptor)” OR “PDGFR (Platelet-Derived Growth Factor Receptor)” OR “VEGFR (Vascular Endothelial Growth Factor Receptor)” OR “FGFR (Fibroblast Growth Factor Receptor)” OR “NGFR (Nerve Growth Factor Receptor)” OR “HGFR (Hepatocyte Growth Factor Receptor)” OR “EPHR (EPH Receptor)” OR “AXLR (AXL Receptor)” OR “CCKR (CCK Receptor)” OR “TIER (TIE Receptor)” OR “RYKR (RYK Receptor)” OR “DDR (Discoidin Domain Receptor)” OR “RETR (RET Receptor)” OR “ROSR (ROS Receptor)” OR “LTKR (Leukocyte Receptor)” OR “ROR (Receptor Tyrosine Kinase Like Orphan Receptor)” OR “MUSKR (Muscle Associated Receptor)” OR “LMR(Lemur Receptor)”].

### Inclusion and exclusion criteria

Studies were included in our meta-analysis and systematic review if they met the following criteria: (i) clinical study of RTK expression in melanoma; (ii) patients were diagnosed with melanoma by pathological or histological examination; (iii) immunohistochemical staining (IHC) was used to detect expression of RTKs in melanoma tissue; (iv) studies provided sufficient survival information for extraction or calculation of the individual Hazard Ratios (HR) and 95% Confidential Intervals (CI). We excluded studies if they met the following exclusion criteria: (i) melanoma was diagnosed without pathological or histological examination; (ii) basic research using cell line or animal model experiment; (iii) duplicate articles; (iv) review, conference abstracts, case reports, and letters. Two trained investigators independently screened study titles, abstracts, and full-text manuscripts for eligibility and disagreements were resolved by consensus of a third investigator.

### Data extraction

Two independent reviewers (PJ and YH) extracted the following data from each selected manuscript: author name, year of publication, country, median patient age, study type, tissue type, RTKs and their expression, antibody used, cut-off value, clinicopathological features, follow-up time, outcome of study (time to event variables), HRs with 95% CIs for survival data, and Kaplan–Meier curves. Survival data were obtained from Kaplan–Meier curves. For studies without HR and 95% CI, we used the methodology previously proposed by Tierney and colleagues (11). Then, a third investigator (JG) verified the accuracy of the synthesized data, and disagreements were resolved by consensus.

### Quality assessment

Quality assessment was performed by two investigators (XL and JG) independently using the 20-item Reporting Recommendations for Tumor Marker Prognostic Studies (REMARK) checklist (12, 13). The detailed explanation of 20 items used the checklist of McShane LM (14). According to the 20 items, each study was characterized as fully satisfied, partially satisfied, not satisfied, unclear, and not applicable. Discrepancies were resolved by a third investigator (LM).

### Statistical analysis

The primary outcomes were Overall Survival (OS), Disease-Free Survival (DFS), and Progression-Free Survival (PFS). HR measuring the association between RTKs and its prognostic data were directly extracted from studies or estimated from the Kaplan–Meier survival curves with their 95% CI. Review Manager 5.3 was used for meta-analysis. Estimates of OS, DFS, or PFS were reported using HR and 95% CI. *I^2^* value was used to describe heterogeneity among studies and P<0.05 indicated statistical significance. Subgroup analyses were used to study the prognostic value of RTKs by clinicopathological features, including disease type, geographic area, and the cut-off for each RTK marker.

## Results

**Figure 1:**
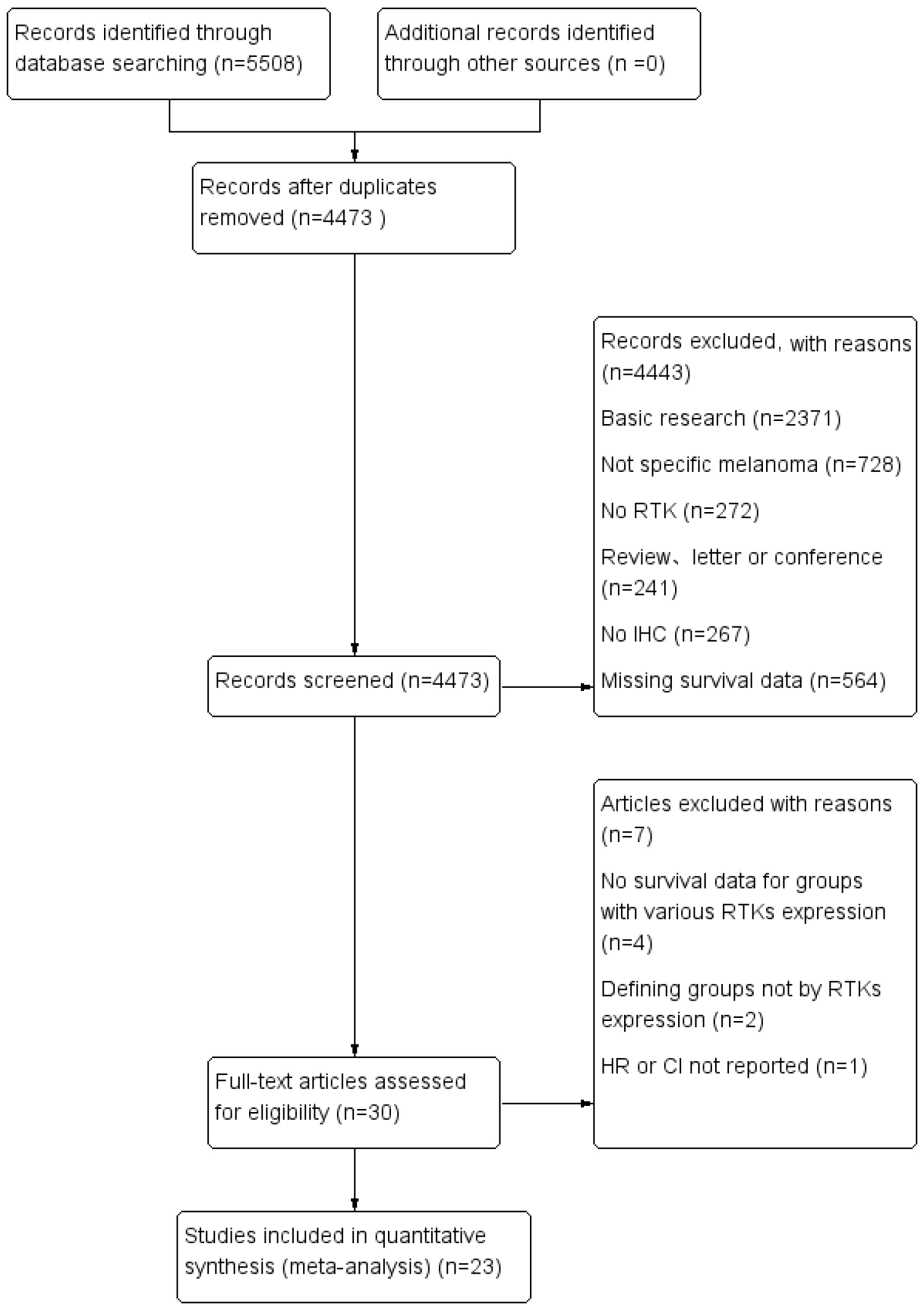
PRISMA flow diagram of selection process.

A total of 5,508 citations were identified from seven electronic databases (886 from PubMed, 74 from Cochrane, 285 from EBSCOhost, 2,234 from Embase, 421 from Ovid, 294 from ScienceDirect, and 1,314 from Web of Science). We excluded 5,478 studies after removing duplicates and screening titles and abstracts based on the exclusion criteria. Subsequently, 30 studies were assessed for eligibility by full-text reviewing. Among these studies, four studies were excluded due to the lack of sufficient survival data, two studies were excluded for not defining groups by RTKs expression and one was excluded because the HR or CI was not reported. Finally, 23 studies met the inclusion criteria and were selected for this meta-analysis. Among the included studies, eight studies used the Tierney method to estimate survival data from Kaplan–Meier curves due to the lack of direct survival data. The flow diagram shown in **Figure 1** depicts the complete selection process.

### Study characteristics

The characteristics of 23 studies are presented in **Table 1**, which includes a total of 2,072 patients (15–37). Sample sizes ranged from 10 to 238. A total of 12 different RTKs were evaluated: EGFR, human epidermal growth factor receptor (HER)2, HER3, HER4, IGF-1R, VEGF-R1, VEGF-R2, VEGF-R3, mesenchymal-epithelial transition factor (MET), C-KIT, EphrinA1, and EphA2. RTK relative expression, antibodies used, and cut-off of biomarkers in each study are detailed in **Table 2**.

**Table 1:** Characteristics of included studies.

Author	Year	Country	Case	Age	Breslow thickness	Metastasis	Disease type	Follow-up	Outcome	Significant findings
Al-Jamal	2011	Finland	167	NG	NG	53 (29.52%)	uveal melanoma	20 years (16-25)	OS	IGF-IR did not independently predict metastasis from primary uveal melanoma.
Boone	2011	Belgium	114	52 years (37–64)	NG	25 (21.9%)	melanoma	33 months (17–50)	OS, DFS	EGFR involves in progression and metastasis of a subset of melanomas.
Chen	2012	China	56	44 ± 2 years (18-78)	NG	5 (8.93%)	uveal melanoma	45.8 ± 3.0 months (6-156)	OS	Overexpression of EphA2 is correlated with prognosis of choroidal melanoma.
Das	2019	Sweden	40	64 years (42–86)	NG	NG	cutaneous melanoma	NG	OS	Higher MET expression had a shorter OS in cutaneous melanoma.
Economou	2005	Sweden	132	63 years (25–85)	NG	55 (41.67%)	uveal melanoma	NG	OS	IGF-1R may play as a prognostic role in uveal melanoma.
Eliopoulos	2002	UK	51	NG	≥10 mm 51 ≤1 mm11	15 (29.41%)	melanoma	NG	OS	HER-2 overexpression has no prognostic significance in thick melanoma.
Ericsson	2002	Sweden	36	61 years (23-87)	NG	18 (50%)	uveal melanoma	138.25 ± 90.99 months (1-245)	OS	High IGF-1R expression is a predictor for the metastasis of uveal melanoma:
Giatromanolaki	2012	Greece	60	NG	≤8 mm 26(43.33%) >8 mm 34(56.67%)	NG	uveal melanoma	80 months (1-154)	OS	pVEGFR2/KDR was significantly related with poor prognosis of uveal melanoma.
Hurks	2000	Netherland	22	66 years (38-91)	NG	7 (31.82%)	uveal melanoma	NG	OS	EGFR expression is an important prognostic factor in human uveal melanoma.
Jafari	2018	Switzerland	238	62.3 ± 15.8 years	2.3 ± 2.7 mm	19 (25.3%)	melanoma	5.71 years	OS, DFS	VEGF-C and VEGF-R2 might be new prognostic marker in melanoma.
Katunarić	2014	Croatia	110	52.25 years (31–79)	3.8 mm (0.8–15)	NG	melanoma	NG	OS	EGFR protein overexpression is correlated with shorter OS in melanoma.
Langer	2011	Germany	10	65 years (55–75)	NG	NG	esophageal melanoma	NG	OS	Esophageal melanomas harbor genetic aberrations of c-Kit, KRAS, and BRAF.
Liu	2008	China	56	56.05 ± 11.34 years (27–81)	1.83 ± 1.03 mm (0.3–4.1)	31 (55.36%)	melanoma	NG	OS, DFS	VEGF-C and VEGF-D may be indicators for prognostic evaluation of melanoma.
Mallikarjuna	2007	India	60	45 years (9-74)	NG	6 (10%)	uveal melanoma	28.2± 32.44 months	OS	High c-Met expression is associated with death due to uveal melanoma.
Mo	2020	China	91	NG	NG	NG	melanoma	NG	OS	EphA2-high/ephrinA1-low exhibited poorer outcomes than EphA2-high/ephrinA1-high in melanoma
Monteiro	2019	Germany	NG	NG	NG	NG	melanoma	NG	OS	High expression of VEGFR-3 is associated with poor OS in melanoma.
Nielsen	2014	Belgium	105	52 years (25–87)	2.3 mm (0.7–45.0)	105 (100%)	melanoma	NG	PFS	HER4 is associated with PFS of malignant melanoma.
Potti	2004	USA	202	57 years (15–101)	2.6 mm (0.4-8)	NG	melanoma	NG	OS	Both c-Kit and VEGF may have significant therapeutic implications in melanoma.
Reschke	2008	Germany	130	19-90 years	range 0.4-17 mm	53 (40.77%)	cutaneous melanoma	56 ± 25 months	OS	HER3 is a determinant for poor prognosis in melanoma.
Straume	2002	Norway	176	NG	NG	56 (31.82%)	recurrent melanoma	76 months (13-210)	OS	Ephrin-A1/EphA2 pathway might be important for patient survival of melanoma.
Trocmé	2012	Sweden	128	63 ± 11.9 years	NG	58 (45%)	uveal melanoma	NG	OS	Nuclear HER3 is associated with favorable overall survival in uveal melanoma.
Yoshida	2014	USA	24	60.58 ± 14.89 years	NG	24 (100%)	Metastatic uveal melanoma	NG	OS	IGF-1R expression is correlated with poor prognosis in metastatic uveal melanoma.
Zhu	2018	China	64	62 years (27–85)	NG	NG	mucosal melanoma	NG	OS	Positive HER4 expression is correlated with the prognosis in mucosal melanoma.

NG, not given.

**Table 2:** Expression of RTKs in studies.

Author	RTK	Antibody used for evaluation	Cut-off	RTK overexpression
Al-Jamal	IGF-1R	N-20; sc-712, Santa Cruz Biotechnology, Calif; dilution 1:500	≥ 15%	88 (68%)
Boone	EGFR	Zymed Laboratories Inc, CA, USA	≥ 10%	13 (11.4%)
Chen	EphA2	Santa Cruz, USA; dilution 1:200	moderate to strong staining	21 (62.5%)
Das	MET ERBB3	ERBB3: Cell Signaling Technologies; dilution 1:250 MET: Cell Signaling Technologies; dilution 1:300	≥ 20%	ERBB3 12 (92%) MET 9 (43%)
Economou	c-Met IGF-1R	IGF-1R: N-20, Santa Cruz Biotechnology, Inc. (Santa Cruz, CA) c-Met: ImmunKemi (Novocastra Ltd., Newcastle-upon-Tyne, UK)	≥ 10%	c-Met:75 (56.82%) IGF-1R:42 (31.82%)
Eliopoulos	HER2	DAKO Ltd, Cambridgeshire, UK	≥ 10%	15 (29.41%)
Ericsson	IGF-1R	Oncogene Science (Manhasset, NY); dilution 1:1000	≥ 50%	15 (41.67%)
Giatromanolaki	VEGFR2	34a; Oxford University, UK	≥50%	14 (23.3%)
Hurks	EGFR	R-1; Santa Cruz Biotechnology, Santa Cruz, CA; dilution 1:20	NG	6 (28.57%)
Jafari	VEGF-R1 VEGF-R2 VEGF-R3	R&D systems	NG	VEGF-R1 22 (52%) VEGF-R2 68 (57.3%) VEGF-R3 34 (52.7%)
Katunarić	EGFR	Membrane EGFR (Dako) nuclear EGFR (Leica Microsystems)	≥ 10%	NEGFR 24 (21.82%) MEGFR 31 (28.18%)
Langer	C-KIT PDGFR-A	C-KIT: A4502; Dako, Glostrup, Denmark PDGFR-A: 3164; Cell Signaling Technologies, Beverly, MA, USA	intensity > 1+	C-KIT 8 (80%) PDGFR-A 0
Liu	VEGFR-3	Santa Cruz Biotechnology, Inc., Santa Cruz, CA; dilution 1:200	≥ 10% of tumor cells ≥ 5% in endothelial cells	34 (60.71%)
Mallikarjuna	EGFR c-met	EGFR (R-1; 200 μl/ml) c-Met (DQ-13; 100 μg/ml) Santa Cruz Biotechnology, CA, USA	> 10%	EGFR 18 (30%) c-met 33 (55%)
Mo	EphrinA1 EphA2	NG	NG	EphA2 26 (28.6%) ephrinA1 28 (30.8%)
Monteiro	VEGFR-3	NG	NG	NG
Nielsen	HER-4	RB-9045-P1; Thermo Scientific; dilution 1:50	NG	NG
Potti	HER-2/neu c-Kit	A4502; IMPATH, Calif., USA	≥2+ or greater Immunostaining	HER-2/neu 2 (0.9%) c-Kit 46 (22.8%)
Reschke	HER3	clone C-17; Santa Cruz; dilution 1:50	German immunohistochemical scoring (GIS) > 6	moderate to high 85 (65%) high in metastases 35 (40%)
Straume	Ephrin-A1 EphA2	Ephrin-A1: pAb SC-911; Santa Cruz EphA2: pAb SC-924; Santa Cruz	staining index = 9	FGFR 17 (11.7%) Ephrin-A1 23 (15.8%) EphA2 23 (15.9%)
Trocmé	HER3	clone C-17; Santa Cruz; dilution 1:50	‘‘2,’’ strong staining intensity	42 (33%)
Yoshida	IGF-1R	Ventana Medical Systems	3+ staining intensities >85% percentages of positive cells	17 (70.83%)
Zhu	HER4	clone: PC100; Vebdor: Thermo Fisher Scientific Co., (Waltham, Massachusetts, USA); dilution 1: 300	positive tumor cells (Range: 0–100%)	45 (70.3%)

NG, not given.

### Quality of eligible studies

The REMARK checklist is widely used as a guideline to analyze the reporting of tumor markers in prognostic studies. In general, the overall quality of the 23 included studies was relatively high based on the REMARK checklist (**Table S1**), and the detailed clarification of 20 items followed the McShane LM checklist (**Table S2**) (14). Most studies failed to provide the rationale for their sample size, investigate assumptions, conduct sensitivity analyses, and conduct internal validation. In addition, due to the lack of standard prognostic markers recognized by the public, none of the studies showed a comparison of RTK expression with such indicators. Several studies did not clearly define all endpoints and missed estimated effects in multivariable analyses (15, 17, 19, 22, 25, 28, 29). However, because most included studies were retrospective and fulfilled the majority of our criteria, they have provided sufficient and convincing data for a comprehensive analysis.

### Association between RTKs and OS

**Figure 2:**
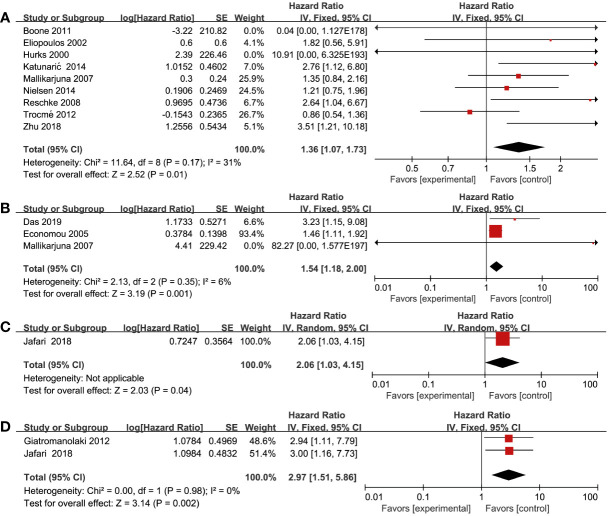
Forest plot illustrating the association between various RTKs and OS in melanoma. **(A)** EGFR, **(B)** MET, **(C)** VEGF-R1, **(D)** VEGF-R2.

All included studies reported on the correlation between RTKs and OS (15–37). From these studies, we found that there was an association between overexpression of RTKs and OS. Worse survival could be found in patients with overexpression of EGFR (HR = 1.36; 95% CI, 1.07-1.73, P = 0.01, I^2^ = 31%), MET (HR = 1.54; 95% CI, 1.18-2.00, P = 0.001, I^2^ = 6%), VEGF-R1 (HR = 2.06; 95% CI, 1.03-4.15, P = 0.04), and VEGF-R2 (HR = 2.97; 95% CI, 1.51-5.86, P = 0.002, I^2^ = 0%) (**Figure 2**). However, there was no statistical difference between OS and IGF-1R (HR = 1.31; 95% CI, 0.92-1.87, P = 0.13, I^2^ = 88%), VEGF-R3 (HR = 1.76; 95% CI, 0.99-3.14, P = 0.05, I^2^ = 69%), C-KIT (HR = 0.65; 95% CI, 0.32-1.34, P = 0.24, I^2^ = 48%), EphrinA1 (HR = 1.38; 95% CI, 0.20-9.40, P = 0.74, I^2^ = 92%), and EphA2 (HR = 2.95; 95% CI, 0.84-10.30, P = 0.09, I^2^ = 85%) (**Figure S1**). Sensitivity analysis showed that there was a statistical difference between OS and IGF-1R using a fixed effects model (HR = 1.50; 95% CI, 1.31-1.73, P < 0.00001) without heterogeneity after excluding one study by Al-Jamal et al. (15). Furthermore, we discovered that there existed a statistical difference of pooled effect with no heterogeneity between VEGF-R3 and OS (HR = 2.46; 95% CI, 1.45-4.19, P = 0.0009) after excluding one study by Monteiro et al. (29) by using a fixed effects model.

### Association between RTKs and DFS and PFS

**Figure 3:**
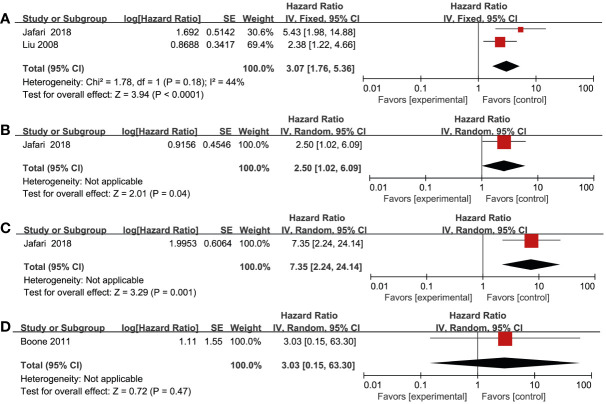
Forest plot illustrating the association between various RTKs and DFS in melanoma. **(A)** VEGF-R3, **(B)** VEGF-R1, **(C)** VEGF-R2, **(D)** EGFR.

Three studies reported DFS as the outcome, which included a total of 408 patients (17, 26, 33). Two studies (26, 33) found a significant association between increased VEGF-R3 and worse DFS in melanoma patients (HR = 3.07; 95% CI, 1.76-5.36, P < 0.0001, I^2^ = 44%) (**Figure 3A**). In addition, there was a significantly worse DFS in patients with overexpression of VEGF-R1 (HR = 2.50; 95% CI, 1.02-6.09, P = 0.04) and VEGF-R2 (HR = 7.35; 95% CI, 2.24-24.14, P = 0.001) (**Figures 3B,C**). However, one study by Boone et al. (17) reported that no significant association in patients with EGFR overexpression (HR = 3.03; 95% CI, 0.15-63.30, P = 0.47). One study by Nielsen et al. (30) found that there was no statistically significant association between high HER-4 and worse PFS (HR = 1.21; 95% CI, 0.75-1.95, P = 0.43) (**Figure S2**).

### Association between RTKs and clinicopathological features

**Figure 4:**
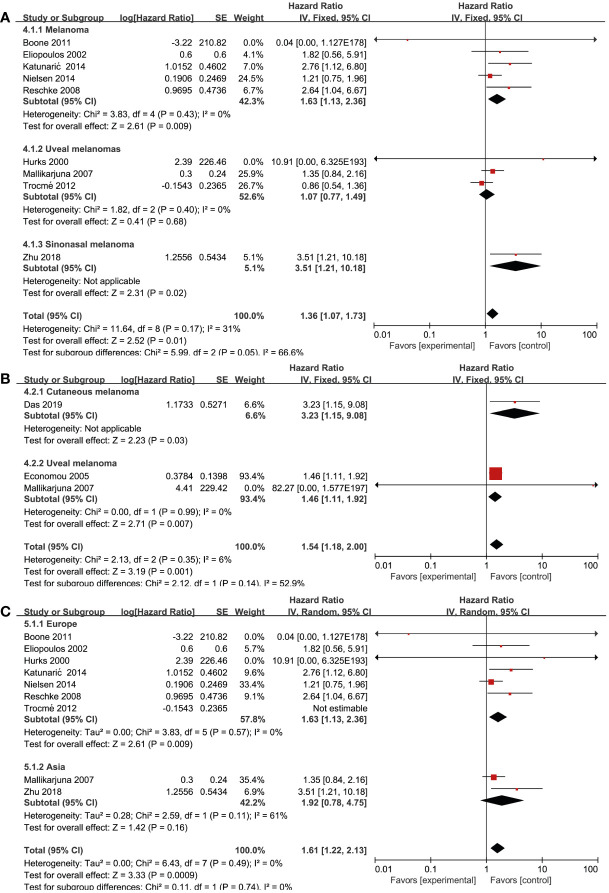
Prognosis of various RTK and clinicopathological features. **(A)** EGFR and disease type, **(B)** MET and disease type, **(C)** EGFR and geographical areas.

Nine studies (17, 21, 23, 24, 27, 30, 32, 35, 37) reported on EGFR and OS. Among them, five (17, 21, 24, 30, 32) reported on cutaneous melanoma, three (23, 27, 35) reported on uveal melanoma, and one (37) reported on nasal melanoma. We performed a subgroup analysis to assess whether the prognostic value of RTKs was related to pathology. By using a fixed effects model, we conducted a pooled analysis from six studies (17, 21, 24, 30, 32, 37), which demonstrated that EGFR overexpression was associated with significantly worse OS in patients with cutaneous melanoma (HR = 1.63; 95% CI, 1.13-2.36, P = 0.009, I^2^ = 0%) and nasal melanoma (HR = 3.51; 95% CI, 1.21-10.18, P = 0.02). However, there were no significant association between EGFR overexpression and uveal melanoma (HR = 1.07; 95% CI, 0.77-1.49, P = 0.68, I^2^ = 0%) (**Figure 4A**). Three studies (19, 20, 27) reported on the association between pathology and MET expression. MET overexpression was associated with a worse OS in cutaneous melanoma (HR = 3.23; 95% CI, 1.15-9.08, P = 0.03) and uveal melanoma patients (HR = 1.46; 95% CI, 1.11-1.92, P = 0.007, I^2^ = 0%) using a fixed effects model (**Figure 4B**). To find whether the prognostic value of RTKs is related to geographic research area, we performed a subgroup analysis for various categories: Europe, America, and Asia. Pooled analysis of EGFR expression from seven studies (17, 21, 23, 24, 27, 30, 32, 35, 37) demonstrated that EGFR overexpression was associated with a worse OS in Europe Genesis(HR = 1.41; 95% CI, 0.95-2.10, P = 0.09, I^2^ = 28%) and Asia (HR = 1.92; 95% CI, 0.78-4.75, P = 0.16, I^2^ = 61%) compared to other geographic areas (**Figure 4C**). After excluding one study by Trocme et al. (35), a statistically significant association was found in European patients with EGFR overexpression (HR = 1.63; 95% CI, 1.13-2.36, P = 0.009, I^2^ = 0). However, we could not study the overall effect of other RTKs due to the lack of sufficient studies and huge heterogeneity within the limited studies.

### Association between RTKs and biomarker cut-off

**Figure 5:**
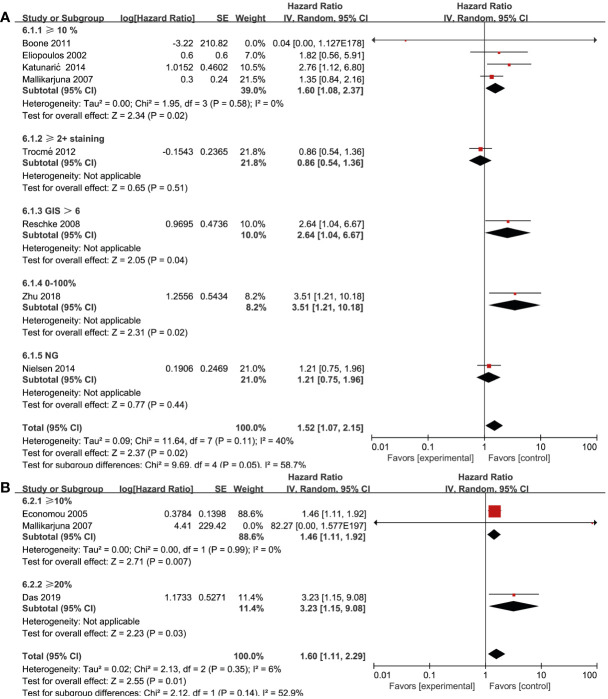
Association of various RTKs and cut-off. **(A)** EGFR, **(B)** MET.

Biomarker cut-offs represented an important source of heterogeneity. Among the eight studies (17, 21, 24, 27, 30, 32, 35, 37) that reported on EGFR and OS, four (17, 21, 24, 27) of them used “≥10% of the tumor” as the cut-off, one (35) used “≥2+ staining”, one (37) used “0–100% staining”, one (32) used “German immunohistochemical scoring (GIS)>6”, and one (30) did not provide a clear definition. The study that used a cut-off of “≥10% of the tumor” revealed a significant association between EGFR expression and OS (HR = 1.60; 95% CI, 1.08-2.37, P = 0.02, I^2^ = 0%), whereas the rest studies did not show strong power due to the limited study quantity (**Figure 5A**). Three studies (19, 20, 27) reported the cut-offs for MET expression: two of them (20, 27) used “≥10%” and the other one (19) used “≥20%”. A statistically significant association was found in both two cut-off categories (“≥10%”, HR = 1.46; 95% CI, 1.11-1.92, P = 0.007, I^2^ = 0%) (**Figure 5B**). Due to the lack of studies focusing on other RTKs and biomarker cut-offs, we could not measure the pooled effect of these variables.

## Discussion

To our knowledge, this is the first and largest meta-analysis that systematically explores the prognostic value of RTKs in malignant melanoma, which included 23 studies with a total of 2,072 patients. Our findings suggest that overexpression of RTKs, based on IHC analysis, is closely associated with poor prognosis in malignant melanoma patients. Furthermore, the prognostic value of the examined RTKs varied according to the clinicopathological characteristics of patients, such as pathological subtype, geographical area, and cut-offs of biomarkers, highlighting the clinical and predictive value of RTK expression.

The pooled prognostic value of RTK overexpression in melanoma has major implications for the field with respect to accurate survival estimation and the selection of individualized combination therapies. By comprehensively gathering and evaluating studies utilizing IHC analysis for resected melanoma, we innovatively investigated the relationship between overexpression of RTKs and survival outcomes. Our results indicated the prognostic value of overexpression of RTKs, including EGFR, MET, VEGF-R1, VEGF-R2, VEGF-R3, and IGF-1R. Numerous studies have reported that aberrant overexpression of RTKs were related with the pathogenesis of melanoma and these RTKs might be used as therapeutic targets. The abnormal expression and activation of EGFR are closely related to the progression and drug resistance of melanoma patients (38, 39). In our study, we also found an association between EGFR overexpression and worse OS in melanoma patients. Additionally, VEGFR has been identified as a potential therapeutic target for the treatment of melanoma, which may inhibit malignant melanoma metastasis and progression. Furthermore, several VEGFR inhibitors have been used in clinical trials to treat melanoma patients (40–42). Roger et al. found VEGFR expression can be used to evaluate chemotherapy efficacy and prognosis of melanoma patients following chemotherapy treatment (43). Our findings are consistent with their conclusions as the pooled HRs of survival data concerning VEGFR overexpression are relatively higher than other RTKs. Hepatocyte growth factor receptor (c-mesenchymal-epithelial transition factor, c-Met) is a transmembrane protein encoded by the Mesenchymal-epithelial transition factor (Met) gene, which is usually abnormally expressed in melanoma due to increased copy number, exon skipping, and gene mutations (19, 44). Several studies also found that c-MET may represent a potential biomarker and therapeutic target for melanoma, which warrants further exploration (45, 46). We also found that MET overexpression is associated with worse OS in melanoma patients, which could be partly explained by the oncogenic role of the Met pathway in the process of drug resistance and immune response. In addition, Villanueva et al. observed that the increased IGF-1R in post-relapse melanoma is consistent with acquired BRAF inhibitors resistance, which also confirmed the prognostic value of IGF-1R in disease progression (47). With more and more clinical trials targeting RTKs, the prognostic value of RTKs and combined therapies are expected to bring new hope to advanced melanoma patients.

In this meta-analysis, the association between the prognostic value of RTK overexpression and the clinicopathological characteristics of melanoma, including pathological subtype, geographic area, and the cut-offs for IHC analysis, was also explored. RTK expression or mutations depends on the melanoma subtype, such as mucosal melanoma (vs. cutaneous melanoma), acral lentiginous melanoma (vs. other cutaneous melanoma), and amelanotic melanoma (vs. melanotic melanoma). Due to the heterogeneity of melanoma, it is critical to investigate relevant RTKs based on their expression and prognostic value by disease subtype. By utilizing subgroup analysis, we found EGFR overexpression was associated with worse OS in cutaneous melanoma and nasal sinus melanoma, but not uveal melanoma. Moreover, MET overexpression was associated with worse OS in both cutaneous melanoma and uveal melanoma. Topcu-Yilmaz et al. suggested that EGFR overexpression was significantly correlated with clinicopathological parameters, such as mitosis rate, in uveal melanomas (48). We believe that the difference may be related to the different evaluating outcomes given we focused on survival data such as OS, PFS, and DFS. In addition, c-Kit mutations and expression were found in mucosal melanoma, acral lentiginous melanoma, and amelanotic melanoma. However, there was no significant association between OS and c-KIT in our study, which might be attributed to melanoma anatomical heterogeneity.

The incidence and prognosis of melanoma patients from various geographic regions were quite different. For instance, the proportion of acral melanoma in black patients with cutaneous melanoma was 80.0%, whereas it was relatively infrequent in Caucasian patients (49, 50). Furthermore, African descendants had more advanced disease stages and higher melanoma-specific mortality compared to Caucasians who usually had a better prognosis (51–53). In our study, we found a statistically significant association between EGFR expression and patients in Europe compared to other geographic areas. However, due to a lack of enough studies on these markers, we could not conduct a comprehensive analysis on the relationship between other RTKs and geographic factors, which might affect the geographic location-specific clinical application of RTK biomarkers for prognostic prediction.

The major strength of our study was the overall prognostic analysis of RTKs and their connection with clinicopathological characteristics. We strictly evaluated the quality of all included studies using the REMARK guidelines. We found some reports did not clearly define all endpoints and overlooked estimated effects in multivariable analyses, which were excluded from our analysis. Furthermore, we explored heterogeneity due to varying biomarker cut-offs used in different studies, which may directly influence the definition of RTK overexpression. We found that studies with EGFR or MET overexpression showed significant prognostic value in patients when the cut-off “≥10% staining of tumor cells” was applied. However, some included studies did not define the specific cut-off or used different cut-off standards from staining scores or other evaluation scores such as GIS scores. Future studies should unify on the cut-offs of biomarkers to conduct homogeneous research. Besides, single-target therapies are often ineffective and prone to recurrence in cancer treatment (54). Currently, most studies focusing combining targeting RTKs with immunotherapy are confined to basic studies, although several therapies using multi-target TKIs, such as imatinib and ipilimumab, have entered clinical trials (55). Due to the existing diversity in patients’ genetic subtypes and pathological characteristics, targeting prognostic RTKs with combination therapies may provide a comprehensive treatment regimen which may produce a long-term therapeutic effect and reduce immune-related adverse events.

This meta-analysis suffers from several limitations. First, due to the lack of sufficient studies reporting clinicopathology issues, such as recurrence, invasion (Breslow thickness), and distant metastasis, we could not conduct a comprehensive analysis on the relationship between these clinicopathologic variables and prognosis or survival. Also, we could not measure the publication bias due to the limited number of studies on each outcome. Additionally, some heterogeneity may arise due to the fact that survival data from several studies were estimated from Kaplan–Meier curves, which increased the chances of deviation to some extent. Most cases were retrospective analyses rather than randomized controlled clinical trials or prospective cohort studies, which may lead to publication bias. Finally, some RTKs have been studied extensively, whereas others are disadvantaged by limited studies. Such analysis can serve as preliminary findings on these lesser studied RTKs, although studies with large sample sizes are needed to get much more data to draw reliable conclusions.

In conclusion, our study provides concrete evidence that overexpression of RTKs is associated with poor prognosis and clinicopathology in malignant melanoma, highlighting the value of RTK in individualized combination therapies and accurate prognostic evaluation. The standard evaluating procedures and proper patients based on RTK expression should be further investigated. Randomized controlled trials or prospective cohort studies with large sample sizes are still required to comprehensively improve the prognostic application and combination therapies targeting RTKs in cancer research.

## Data availability statement

The original contributions presented in the study are included in the article/**Supplementary Material**. Further inquiries can be directed to the corresponding author.

## Author contributions

XL, YZ and NT contributed to conception and design. XL, LM and PJ contributed to the methodology. XL, YZ and LM searched the literature. PJ, YH and JG extracted the data and conducted the statistical analysis. XL, JG and LM contributed the quality assessment. XL, YZ and LM wrote the manuscript. NT revised the manuscript. All authors contributed to the article and approved the submitted version.

## Funding

This work was supported by the Postgraduate Practice Innovation Program of Jiangsu Province (SJCX20_1434).

## Conflict of interest

The authors declare that the research was conducted in the absence of any commercial or financial relationships that could be construed as a potential conflict of interest.

## Publisher’s note

All claims expressed in this article are solely those of the authors and do not necessarily represent those of their affiliated organizations, or those of the publisher, the editors and the reviewers. Any product that may be evaluated in this article, or claim that may be made by its manufacturer, is not guaranteed or endorsed by the publisher.

## References

[1] Welch HG Mazer BL Adamson AS . The rapid rise in cutaneous melanoma diagnoses. New Engl J Med (2021) 384:72–9. doi: 10.1056/NEJMsb2019760 33406334

[2] Versluis JM Hendriks AM Weppler AM Brown LJ de Joode K Suijkerbuijk KPM . The role of local therapy in the treatment of solitary melanoma progression on immune checkpoint inhibition: A multicentre retrospective analysis. Eur J Cancer (Oxford Engl (2021) 1990):151. doi: 10.1016/j.ejca.2021.04.003 33971447

[3] Aldonza MBD Ku J Hong JY Kim D Yu SJ Lee MS . Prior acquired resistance to paclitaxel relays diverse EGFR-targeted therapy persistence mechanisms. Sci Adv (2020) 6:eaav7416. doi: 10.1126/sciadv.aav7416 32083171 PMC7007258

[4] Saraon P Snider J Kalaidzidis Y Wybenga-Groot LE Weiss K Rai A . A drug discovery platform to identify compounds that inhibit EGFR triple mutants. Nat Chem Biol (2020) 16:577–86. doi: 10.1038/s41589-020-0484-2 PMC8123931 32094923

[5] Zhang Y Liu S Zhou S Yu D Gu J Qin Q . Focal adhesion kinase: Insight into its roles and therapeutic potential in oesophageal cancer. Cancer Lett (2021) 496:93–103. doi: 10.1016/j.canlet.2020.10.005 33038490

[6] Pietrobono S Anichini G Sala C Manetti F Almada LL Pepe S . ST3GAL1 is a target of the SOX2-GLI1 transcriptional complex and promotes melanoma metastasis through AXL. Nat Commun (2020) 11:5865. doi: 10.1038/s41467-020-19575-2 33203881 PMC7673140

[7] Faião-Flores F Emmons MF Durante MA Kinose F Saha B Fang B . HDAC inhibition enhances the *In vivo* efficacy of MEK inhibitor therapy in uveal melanoma. Clin Cancer Res (2019) 25:5686–701. doi: 10.1158/1078-0432.ccr-18-3382 PMC6744978 31227503

[8] Greenhalgh J Boland A Bates V Vecchio F Dundar Y Chaplin M . First-line treatment of advanced epidermal growth factor receptor (EGFR) mutation positive non-squamous non-small cell lung cancer. Cochrane Database Syst Rev (2021) 3:Cd010383. doi: 10.1002/14651858.CD010383.pub3 33734432 PMC8092455

[9] Cascone T Xu L Lin HY Liu W Tran HT Liu Y . The HGF/c-MET pathway is a driver and biomarker of VEGFR-inhibitor resistance and vascular remodeling in non-small cell lung cancer. Clin Cancer Res (2017) 23:5489–501. doi: 10.1158/1078-0432.ccr-16-3216 PMC5600821 28559461

[10] Guo R Luo J Chang J Rekhtman N Arcila M Drilon A . MET-dependent solid tumours - molecular diagnosis and targeted therapy. Nat Rev Clin Oncol (2020) 17:569–87. doi: 10.1038/s41571-020-0377-z PMC7478851 32514147

[11] Tierney JF Stewart LA Ghersi D Burdett S Sydes MR . Practical methods for incorporating summary time-to-event data into meta-analysis. Trials (2007) 8:16. doi: 10.1186/1745-6215-8-16 17555582 PMC1920534

[12] McShane LM Altman DG Sauerbrei W Taube SE Gion M Clark GM . REporting recommendations for tumor MARKer prognostic studies (REMARK). Nat Clin Pract Oncol (2005) 2:416–22. doi: 10.1038/ncponc0252 16130938

[13] Sauerbrei W Taube SE McShane LM Cavenagh MM Altman DG . Reporting recommendations for tumor marker prognostic studies (REMARK): An abridged explanation and elaboration. J Natl Cancer Institute (2018) 110:803–11. doi: 10.1093/jnci/djy088 PMC6093349 29873743

[14] McShane LM Altman DG Sauerbrei W Taube SE Gion M Clark GM . Reporting recommendations for tumor marker prognostic studies (REMARK). J Natl Cancer Institute (2005) 97:1180–4. doi: 10.1093/jnci/dji237 16106022

[15] Al-Jamal RT Kivelä T . Prognostic associations of insulin-like growth factor-1 receptor in primary uveal melanoma. Can J Ophthalmol (2011) 46:471–6. doi: 10.1016/j.jcjo.2011.09.013 22153631

[16] All-Ericsson C Girnita L Seregard S Bartolazzi A Jager MJ Larsson O . Insulin-like growth factor-1 receptor in uveal melanoma: a predictor for metastatic disease and a potential therapeutic target. Invest Ophthalmol Vis Sci (2002) 43:1–8. 11773005

[17] Boone B Jacobs K Ferdinande L Taildeman J Lambert J Peeters M . EGFR in melanoma: clinical significance and potential therapeutic target. J Cutan Pathol (2011) 38:492–502. doi: 10.1111/j.1600-0560.2011.01673.x 21352258

[18] Chen LX Sun BC Li XR He YJ Song GX . [Overexpression of the receptor tyrosine kinase EphA2 in choroidal melanoma: correlation with vesculogenic mimicry and prognosis]. [Zhonghua yan ke za zhi] Chin J Ophthalmol (2012) 48:985–90. doi: 10.3760/cma.j.issn.0412-4081.2012.11.007 23302271

[19] Das I Wilhelm M Höiom V Franco Marquez R Costa Svedman F Hansson J . Combining ERBB family and MET inhibitors is an effective therapeutic strategy in cutaneous malignant melanoma independent of BRAF/NRAS mutation status. Cell Death Dis (2019) 10:663. doi: 10.1038/s41419-019-1875-8 31506424 PMC6737096

[20] Economou MA All-Ericsson C Bykov V Girnita L Bartolazzi A Larsson O . Receptors for the liver synthesized growth factors IGF-1 and HGF/SF in uveal melanoma: intercorrelation and prognostic implications. Invest Ophthalmol Vis Sci (2005) 46:4372–5. doi: 10.1167/iovs.05-0322 16303922

[21] Eliopoulos P Mohammed MQ Henry K Retsas S . Overexpression of HER-2 in thick melanoma. Melanoma Res (2002) 12:139–45. doi: 10.1097/00008390-200204000-00006 11930110

[22] Giatromanolaki A Sivridis E Bechrakis NE Willerding G St Charitoudis G Foerster MH . Phosphorylated pVEGFR2/KDR receptor expression in uveal melanomas: relation with HIF2α and survival. Clin Exp Metastasis (2012) 29:11–7. doi: 10.1007/s10585-011-9424-6 21984395

[23] Hurks HMH Metzelaar-Blok JAW Barthen ER Zwinderman AH De Wolff-Rouendaal D Keunen JEE . Expression of epidermal growth factor receptor: Risk factor in uveal melanoma. Invest Ophthalmol Visual Sci (2000) 41:2023–7. 10892838

[24] Katunaric M Jurisic D Petkovic M Grahovac M Grahovac B Zamolo G . EGFR and cyclin D1 in nodular melanoma: correlation with pathohistological parameters and overall survival. Melanoma Res (2014) 24:584–91. doi: 10.1097/cmr.0000000000000123 25304234

[25] Langer R Becker K Feith M Friess H Hofler H Keller G . Genetic aberrations in primary esophageal melanomas: molecular analysis of c-KIT, PDGFR, KRAS, NRAS and BRAF in a series of 10 cases. Modern Pathol (2011) 24:495–501. doi: 10.1038/modpathol.2010.220 21131919

[26] Liu BQ Ma J Wai XL Su F Li XM Yang SC . Lymphangiogenesis and its relationship with lymphatic metastasis and prognosis in malignant melanoma. Anatomical Record-Advances Integr Anat Evolutionary Biol (2008) 291:1227–35. doi: 10.1002/ar.20736 18561194

[27] Mallikarjuna K Pushparaj V Biswas J Krishnakumar S . Expression of epidermal growth factor receptor, ezrin, hepatocyte growth factor, and c-met in uveal melanoma: An immunohistochemical study. Curr Eye Res (2007) 32:281–90. doi: 10.1080/02713680601161220 17453948

[28] Mo J Zhao X Dong X Liu T Zhao N Zhang D . Effect of EphA2 knockdown on melanoma metastasis depends on intrinsic ephrinA1 level. Cell Oncol (Dordrecht) (2020) 43:655–67. doi: 10.1007/s13402-020-00511-x PMC12990701 32291572

[29] Monteiro AC Muenzner JK Andrade F Rius FE Ostalecki C Geppert CI . Gene expression and promoter methylation of angiogenic and lymphangiogenic factors as prognostic markers in melanoma. Mol Oncol (2019) 13:1433–49. doi: 10.1002/1878-0261.12501 PMC6547615 31069961

[30] Nielsen TO Poulsen SS Journe F Ghanem G Sorensen BS . HER4 and its cytoplasmic isoforms are associated with progression-free survival of malignant melanoma. Melanoma Res (2014) 24:88–91. doi: 10.1097/cmr.0000000000000040 24366194

[31] Potti A Moazzam N Langness E Sholes K Tendulkar K Koch M . Immunohistochemical determination of HER-2/neu, c-kit (CD117), and vascular endothelial growth factor (VEGF) overexpression in malignant melanoma. J Cancer Res Clin Oncol (2004) 130:80–6. doi: 10.1007/s00432-003-0509-8 PMC12161809 14634801

[32] Reschke M Mihic-Probst D van der Horst EH Knyazev P Wild PJ Hutterer M . HER3 is a determinant for poor prognosis in melanoma. Clin Cancer Res (2008) 14:5188–97. doi: 10.1158/1078-0432.ccr-08-0186 18698037

[33] Seyed Jafari SM Wiedmer C Cazzaniga S Frangež Ž. Shafighi M Beltraminelli H . Correlation of vascular endothelial growth factor subtypes and their receptors with melanoma progression: A next-generation tissue microarray (ngTMA) automated analysis. PLoS One (2018) 13:e0207019. doi: 10.1371/journal.pone.0207019 30408085 PMC6224082

[34] Straume O Akslen LA . Importance of vascular phenotype by basic fibroblast growth factor, and influence of the angiogenic factors basic fibroblast growth factor/fibroblast growth factor receptor-1 and ephrin-A1/EphA2 on melanoma progression. Am J Pathol (2002) 160:1009–19. doi: 10.1016/S0002-9440(10)64922-X PMC1867162 11891198

[35] Trocme E Mougiakakos D Johansson CC All-Eriksson C Economou MA Larsson O . Nuclear HER3 is associated with favorable overall survival in uveal melanoma. Int J Cancer (2012) 130:1120–7. doi: 10.1002/ijc.26118 21484789

[36] Yoshida M Selvan S McCue PA DeAngelis T Baserga R Fujii A . Expression of insulin-like growth factor-1 receptor in metastatic uveal melanoma and implications for potential autocrine and paracrine tumor cell growth. Pigment Cell Melanoma Res (2014) 27:297–308. doi: 10.1111/pcmr.12206 24354797

[37] Zhu W Li S Zou B Liu H Wang S . Expressions and clinical significance of HER4 and CD44 in sinonasal mucosal malignant melanoma. Melanoma Res (2018) 28:105–10. doi: 10.1097/CMR.0000000000000428 29309357

[38] Wang J Huang SK Marzese DM Hsu SC Kawas NP Chong KK . Epigenetic changes of EGFR have an important role in BRAF inhibitor-resistant cutaneous melanomas. J Invest Dermatol (2015) 135:532–41. doi: 10.1038/jid.2014.418 PMC4307785 25243790

[39] Ji Z Erin Chen Y Kumar R Taylor M Jenny Njauw C-N Miao B . MITF modulates therapeutic resistance through EGFR signaling. J Invest Dermatol (2015) 135:1863–72. doi: 10.1038/jid.2015.105 PMC4466007 25789707

[40] Li J Qin S Xu RH Shen L Xu J Bai Y . Effect of fruquintinib vs placebo on overall survival in patients with previously treated metastatic colorectal cancer: The FRESCO randomized clinical trial. JAMA (2018) 319:2486–96. doi: 10.1001/jama.2018.7855 PMC6583690 29946728

[41] Albiges L Barthélémy P Gross-Goupil M Negrier S Needle MN Escudier B . TiNivo: safety and efficacy of tivozanib-nivolumab combination therapy in patients with metastatic renal cell carcinoma. Ann Oncol (2021) 32:97–102. doi: 10.1016/j.annonc.2020.09.021 33010459

[42] Schöffski P Mir O Kasper B Papai Z Blay JY Italiano A . Activity and safety of the multi-target tyrosine kinase inhibitor cabozantinib in patients with metastatic gastrointestinal stromal tumour after treatment with imatinib and sunitinib: European organisation for research and treatment of cancer phase II trial 1317 ‘CaboGIST’. Eur J Cancer (Oxford England: 1990) (2020) 134:62–74. doi: 10.1016/j.ejca.2020.04.021 32470848

[43] Mouawad R Spano JP Comperat E Capron F Khayat D . Tumoural expression and circulating level of VEGFR-3 (Flt-4) in metastatic melanoma patients: correlation with clinical parameters and outcome. Eur J Cancer (2009) 45:1407–14. doi: 10.1016/j.ejca.2008.12.015 19157860

[44] Song KY Desar S Pengo T Shanley R Giubellino A . Correlation of MET and PD-L1 expression in malignant melanoma. Cancers (Basel) (2020) 12(7). doi: 10.3390/cancers12071847 PMC7408820 32659961

[45] Barisione G Fabbi M Gino A Queirolo P Orgiano L Spano L . Potential role of soluble c-met as a new candidate biomarker of metastatic uveal melanoma. JAMA Ophthalmol (2015) 133:1013–21. doi: 10.1001/jamaophthalmol.2015.1766 26068448

[46] Puri N Ahmed S Janamanchi V Tretiakova M Zumba O Krausz T . C-met is a potentially new therapeutic target for treatment of human melanoma. Clin Cancer Res (2007) 13:2246–53. doi: 10.1158/1078-0432.ccr-06-0776 17404109

[47] Villanueva J Vultur A Lee JT Somasundaram R Fukunaga-Kalabis M Cipolla AK . Acquired resistance to BRAF inhibitors mediated by a RAF kinase switch in melanoma can be overcome by cotargeting MEK and IGF-1R/PI3K. Cancer Cell (2010) 18:683–95. doi: 10.1016/j.ccr.2010.11.023 PMC3026446 21156289

[48] Topcu-Yilmaz P Kiratli H Saglam A Söylemezoglu F Hascelik G . Correlation of clinicopathological parameters with HGF, c-met, EGFR, and IGF-1R expression in uveal melanoma. Melanoma Res (2010) 20:126–32. doi: 10.1097/CMR.0b013e328335a916 20061986

[49] De Wet J Tod B Visser WI Jordaan HF Schneider JW . Clinical and pathological features of acral melanoma in a south African population: A retrospective study. South Afr Med J = Suid-Afrikaanse Tydskrif vir Geneeskunde (2018) 108:777–81. doi: 10.7196/SAMJ.2018.v108i9.13435 30182904

[50] Dika E Veronesi G Altimari A Riefolo M Ravaioli GM Piraccini BM . BRAF, KIT, and NRAS mutations of acral melanoma in white patients. Am J Clin Pathol (2020) 153:664–71. doi: 10.1093/ajcp/aqz209 32017841

[51] Shaikh WR Dusza SW Weinstock MA Oliveria SA Geller AC Halpern AC . Melanoma thickness and survival trends in the united states, 1989 to 2009. J Natl Cancer Institute (2016) 108(1). doi: 10.1093/jnci/djv294 PMC4857148 26563354

[52] Anaba EL . Comparative study of cutaneous melanoma and its associated issues between people of African decent and caucasians. Dermatologic Ther (2021) 34:e14790. doi: 10.1111/dth.14790 33480165

[53] Mahendraraj K Sidhu K Lau CSM McRoy GJ Chamberlain RS Smith FO . Malignant melanoma in African-americans: A population-based clinical outcomes study involving 1106 African-American patients from the surveillance, epidemiology, and end result (SEER) database (1988-2011). Med (Baltimore) (2017) 96:e6258. doi: 10.1097/md.0000000000006258 PMC5403065 28403068

[54] Liguoro D Fattore L Mancini R Ciliberto G . Drug tolerance to target therapy in melanoma revealed at single cell level: What next? Biochim Biophys Acta Rev Cancer (2020) 1874:188440. doi: 10.1016/j.bbcan.2020.188440 33007433

[55] Reilley MJ Bailey A Subbiah V Janku F Naing A Falchook G . Phase I clinical trial of combination imatinib and ipilimumab in patients with advanced malignancies. J Immunother Cancer (2017) 5:35. doi: 10.1186/s40425-017-0238-1 28428884 PMC5394629

